# Enhancement of plant cold tolerance by soybean RCC1 family gene *GmTCF1a*

**DOI:** 10.1186/s12870-021-03157-5

**Published:** 2021-08-12

**Authors:** Zhanghui Dong, Hui Wang, Xia Li, Hongtao Ji

**Affiliations:** 1 Shijiazhuang Academy of Agricultural and Forestry Sciences, 479 Shenglibei Street, Shijiazhuang, 050041 Hebei China; 2 National Key Laboratory of Crop Genetic Improvement, College of Plant Science and Technology, Huazhong Agricultural University, Wuhan, 430070 China

**Keywords:** Soybean, Cold tolerance, RCC1-like protein, AtTCF1, GmTCF1a

## Abstract

**Background:**

Low temperature severely limits the growth, yield, and geographic distributions of soybean. Soybean plants respond to cold stress by reprogramming the expression of a series of cold-responsive genes. However, the intrinsic mechanism underlying cold-stress tolerance in soybean remains unclear. *A. thaliana* tolerant to chilling and freezing 1 (AtTCF1) is a regulator of chromosome condensation 1 (RCC1) family protein and regulates freezing tolerance through an independent C-repeat binding transcription factor (CBF) signaling pathway.

**Results:**

In this study, we identified a homologous gene of *AtTCF1* in soybean (named *GmTCF1a*), which mediates plant tolerance to low temperature*.* Like AtTCF1, GmTCF1a contains five RCC1 domains and is located in the nucleus. *GmTCF1a* is strongly and specifically induced by cold stress. Interestingly, ectopic overexpression of *GmTCF1a* in *Arabidopsis* greatly increased plant survival rate and decreased electrolyte leakage under freezing stress. A cold-responsive gene, *COR15a*, was highly induced in the *GmTCF1a*-overexpressing transgenic lines*.*

**Conclusions:**

*GmTCF1a* responded specifically to cold stress, and ectopic expression of *GmTCF1a* enhanced cold tolerance and upregulated *COR15a* levels. These results indicate that *GmTCF1a* positively regulates cold tolerance in soybean and may provide novel insights into genetic improvement of cold tolerance in crops.

**Supplementary Information:**

The online version contains supplementary material available at 10.1186/s12870-021-03157-5.

## Background

As an important cash crop, soybean (*G. max*) provides us not only abundant proteins but also oils for consumption. Soybean is a temperate legume and plants are particularly susceptible to low temperature stress, which significantly limits the growth of soybean and thus severely reduces yield [1]. Soybean seeds and germinating seedlings are extremely sensitive to low temperatures. During the seed germination stage, low temperature affects the germination of soybean seeds and reduces seedling emergence in cold soils by interfering with the normal membrane reorganization during seed imbibition [2]. Low temperatures of about 10 °C or less sustained for an extended period of time can damage the green stems and leaves [3]. During the reproductive stage, the optimum temperatures for seed maturation are 19–20 °C. At this stage, severe chilling stress reduces soybean yield mainly by reducing seed size and delaying maturity [4–6]. Therefore, unravelling the mechanism of how plants avoid low temperature damage could provide valuable information for breeding and improving soybean yield in agricultural production.

Plants have evolved sophisticated mechanisms to cope with ever-changing environments. After a period of exposure to low temperatures (nonfreezing temperatures), plants exhibit greater tolerance to freezing temperature, which is called cold acclimation [7, 8]. Cold acclimation comprises comprehensive physiological, biochemical and molecular actions [9]. At the physiological level, plenty of protective substances including proline, soluble sugars and cold-resistant proteins, etc. are synthesized [10]. At the molecular level, the cold signal is first perceived by cell membrane fluidity, ion channels (i.e., Ca^2+^ channels) and electrophysiology, which then induces plasma membrane rigidification and activates the Ca^2+^ channel, leading to the influx of Ca^2+^ into the cytosol. In *Arabidopsis*, cold signals activate a receptor-like cytoplasmic kinase cold-responsive protein kinase 1 (CRPK1), in which CRPK1 phosphorylates and promotes the accumulation of 14–3-3 proteins in the nucleus. 14–3-3 proteins involve in many physiological processes in plants, including responses to abiotic stress [11]. In the nucleus, phosphorylated 14–3-3 proteins promote the degradation of CBFs via the 26S proteasome [12].

CBFs function as key transcription factors in the nucleus and regulate a complex cold signal transduction network [13]. There are three cold-induced *CBF* genes, *CBF1–3* (*CBF1/DREB1B*, *CBF2/DREB1C* and *CBF3/DREB1A*), arranged in tandem along a chromosome in *Arabidopsis*. The expression of *CBFs* is tightly regulated by several upstream regulators [14]. ICE1 (Inducer of CBF Expression 1), which belongs to basic helix-loop-helix (bHLH) family protein, directly binds to *CBF3* promoter and activates the expression of *CBF3* upon cold stress [15]. The mutation of *ICE1* blocks *CBF3* level and decreases freezing tolerance [15–17]. CBFs regulate 10–20% of downstream COLD REGULATED (*COR*) genes in *Arabidopsis* in responsive to cold treatment [18–20]. *COR* genes contain a dehydration-responsive element/C-repeat (DRE/CRT) *cis*-element as common features in their promoter region. CBFs bind to the DRE/CRT *cis*-elements and activate *COR* genes, which confer chilling and freezing tolerance to plants [21–24]. COR15a is the most distinctive COR protein that localizes at the membrane and maintains the integrity of cell membranes under cold stress [25]. In addition to the CBF-dependent pathway in *Arabidopsis*, *COR* gene is also modulated by several CBF-independent regulators. For example, BRASSINAZOLE RESISTANT1 (BZR1), a transcription factor in the brassinosteroid (BR) signal transduction pathway, modulates plant tolerance to low temperature through other *COR* genes (*PYR1-LIKE 6* (*PYL6*) and *SUPPRESSOR OF OVEREXPRESSION OF CO1* (*SOC1*) uncoupled with CBFs [26]; HOS9, encodes a homeodomain transcription factor, modulates cold signaling through a CBF-independent pathway in *Arabidopsis* [27]. Thus, plant’s response to low temperature is a complex process in *Arabidopsis*.

In soybean, great efforts have been made to understand and improve plant cold tolerance. Proteomic analyses have also revealed that cold-tolerant soybean produces more protective substances [3]. Transcriptome analyses have identified many cold-responsive genes in soybean, including *CBF* genes [28, 29]. Indeed, the ectopic expression of soybean *CBFs* enhanced the freezing tolerance by elevating the levels of *AtCOR47* and *AtRD29a* transcripts in *Arabidopsis* [30]. The *GmDREB3* gene, a member of the DREB A-5 subfamily, has been specifically induced by cold stress, with overexpression of *GmDREB3* in *Arabidopsis* enhancing plant cold tolerance [30, 31]. Several soybean *COR* genes have also been identified as positive regulators of plant cold tolerance [32–35]. For example, Soybean *SCOF-1*, *MYB*, *WRKY*, *bZIP* and Zinc Finger-type transcription factor genes have been shown to mediate plant cold tolerance [32–35]. These preliminary results indicate that soybean has multiple mechanisms to cope with low temperature stress. However, the detailed mechanisms of soybean tolerance to low temperature stress remain largely unknown.

Previously, we identified *AtTCF1* (*Tolerant to Chilling and Freezing 1*) as a *COR* gene in *Arabidopsis*. *AtTCF1* encodes an RCC1-like protein and responds specifically to cold stress by transcription and protein accumulation in *Arabidopsis* [36]. Loss of *AtTCF1* leads to reduced *BCB* (*blue* c*opper-binding*) level and lignin content, results in cold tolerance. The results have revealed the important role of AtTCF1-mediated cell wall remodeling under cold stress. In this study, we identified a putative ortholog of *AtTCF1* in soybean, *GmTCF1a*, which is specifically regulated in response to cold stress. Intriguingly, ectopic expression of *GmTCF1a* in *Arabidopsis* resulted in elevated levels of *COR15a* and enhanced plant tolerance to freezing stress. These results indicate that *GmTCF1a* positively regulates cold tolerance in soybean.

## Results

### Identification of *GmTCF1s* in soybean

**Fig. 1:**
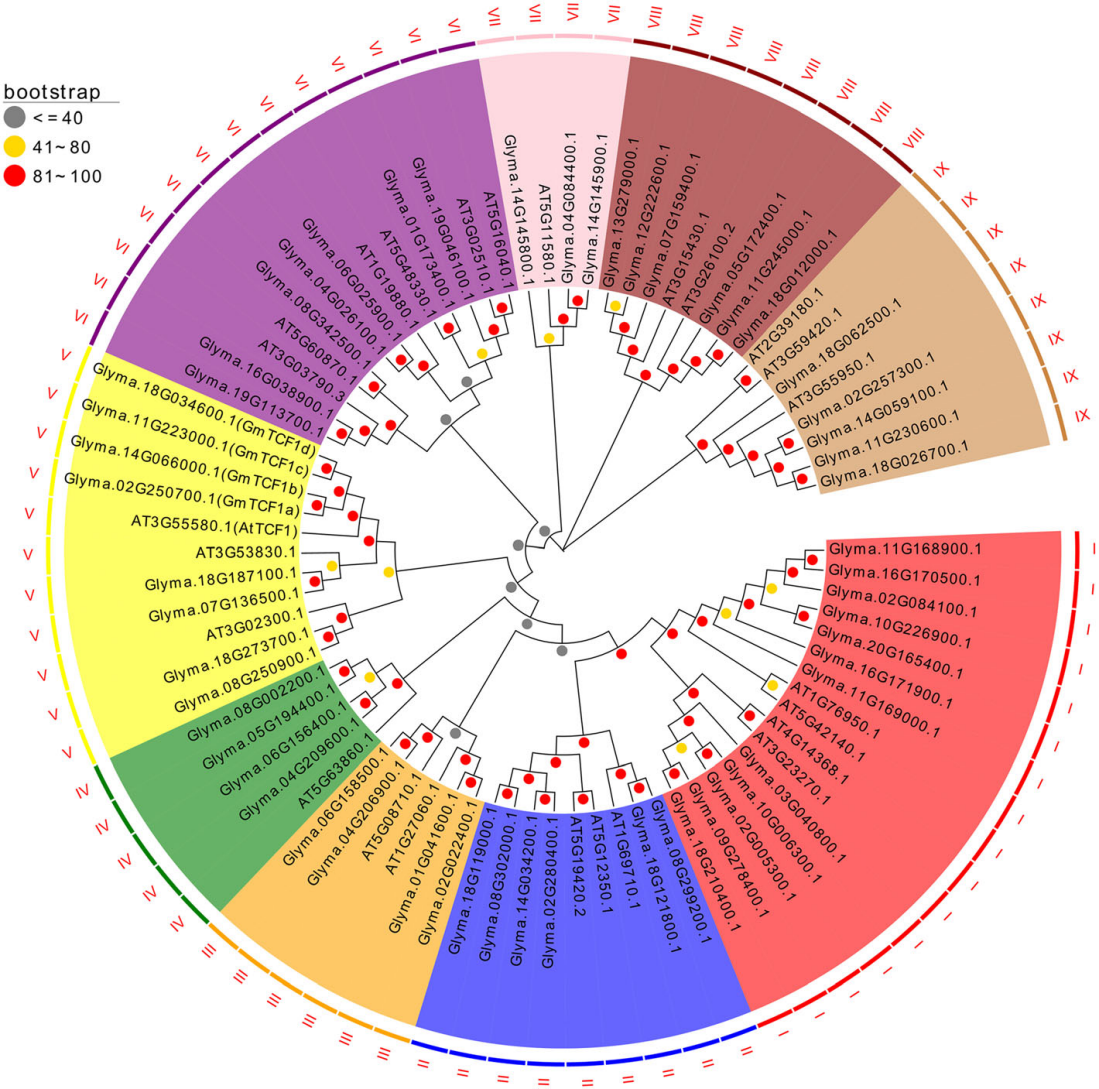
Phylogenetic analysis of RCC1-like proteins from *Glycine max* (Gm) and *A. thaliana* (At). Clustal W was used to align 80 RCC1-like proteins, and MEGA X software was employed to construct a neighbor-joining phylogenetic tree with 1000 bootstrap replications. RCC1-like proteins were separated into nine clades from I to IX. Different clades were distinguished by different colors

To identify homologs of *Arabidopsis TCF1 (AtTCF1)* in soybean, we retrieved protein sequences containing RCC1 domains from *Arabidopsis* and soybean using a HMM (Hidden Markov Model) search (PF00415 and PF13540), and 55 non-redundant soybean genes and 25 non-redundant *Arabidopsis* genes were confirmed as homologs of AtTCF1. A phylogenetic tree was constructed to examine the classification and evolutionary history of these RCC1-like proteins (Fig. 1). All of these RCC1 like proteins were clearly divided into 9 major clades. AtTCF1 and four proteins (Glyma.02G250700, Glyma.14G066000, Glyma.11G223000 and Glyma.18G034600) were grouped in clade V (Fig. 1). This result indicates that AtTCF1 and these four proteins are more closely related than others. Then Glyma.02G250700 was named as GmTCF1a, Glyma.14G066000 as GmTCF1b, Glyma.11G223000 as GmTCF1c and Glyma.18G034600 as GmTCF1d based on the evolutionary relationship with AtTCF1.

**Fig. 2:**
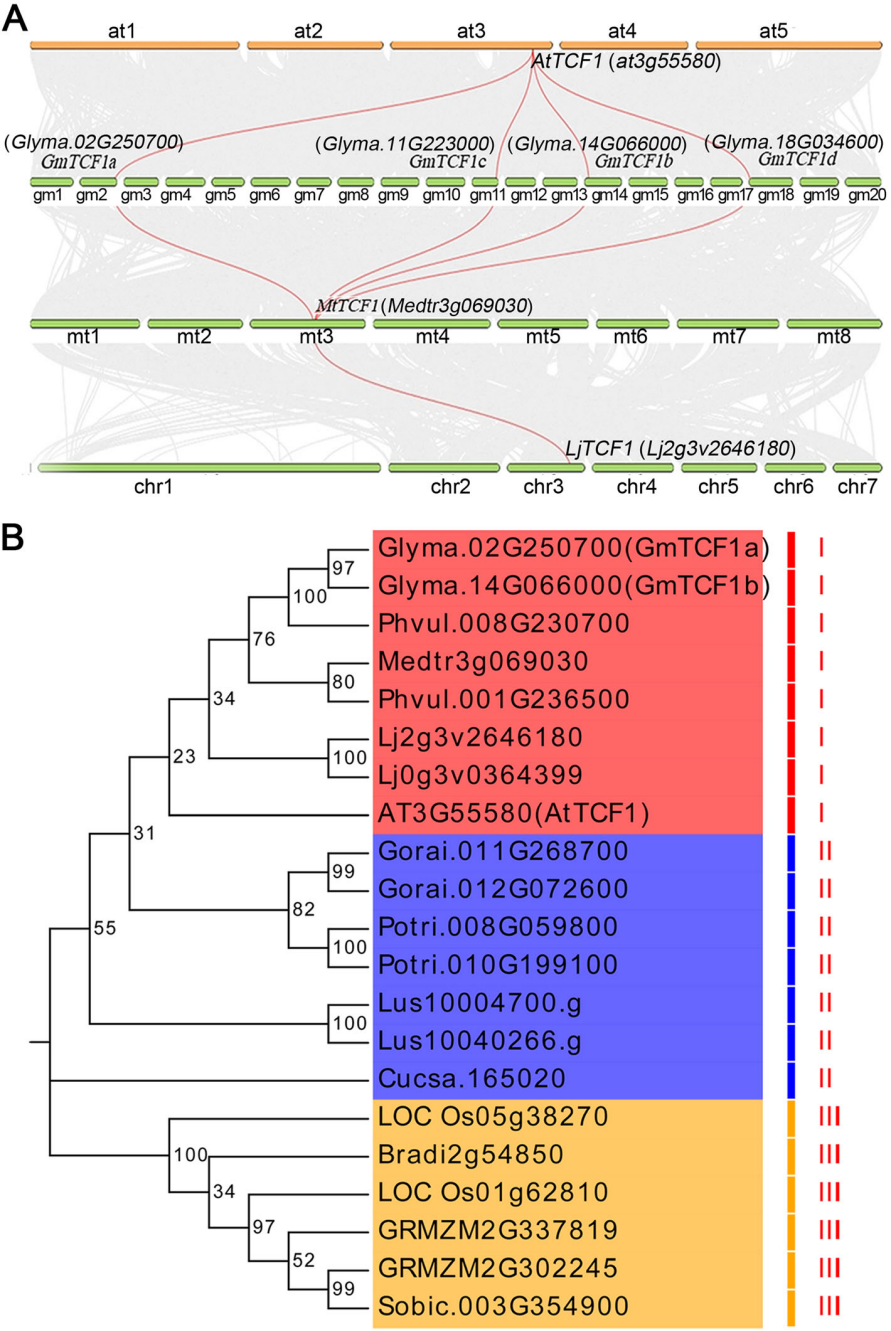
The synteny and phylogenetic analysis of *TCF1* with its homologs. **A** McScanX was used for synteny analysis of *AtTCF1* with homologous genes in *Glycine max*, *Medicago truncatula* and *Lotus japonicus*. Gray lines represent collinear blocks within species, while red lines represent syntenic *TCF1* gene pairs. **B** Phylogenetic tree of GmTCF1s and their homologs from various organisms, including *A. thaliana*, *Oryza sativa*, *Glycine Max*, *Medicago truncatula*, *Lotus japonicus*, *Phaseolus vulgaris*, *Populus trichocarpa*, *Linum usitatissimum*, *Gossypium raimondii*, *Zea mays*, *B. distachyon*, *Sorghum bicolor* and *C. sativus*. Bootstrap support values of 1000 replicates are given at each node. RCC1-like proteins were separated into three clades from I to III

Furthermore, we investigated the synteny of *TCF1* homologous genes between the soybean and *Arabidopsis* genomes. By searching the plant genome duplication database (PGDD), we found that *AtTCF1* displayed collinearity with four *GmTCF1s* genes (*GmTCF1a*, *GmTCF1b*, *GmTCF1b* and *GmTCF1d*) in soybean (Supplementary Fig. S1). Next, we performed a synteny analysis with the *AtTCF1* coding sequence using McScanX in leguminous plants. The same four *GmTCF1s* were found in soybean (Fig. 2a). However, only one collinear gene of *AtTCF1* was found in *Medicago* and *Lotus*, respectively (Fig. 2a). These collinearity results suggest that *AtTCF1* and the four *GmTCF1s* genes may be traced to a common ancestor, and twice the whole genome duplication produced more copies of *AtTCF1* homologous genes in soybean. To explore the evolutionary relationship of TCF1 homologous genes in different species, a phylogenetic tree with 21 TCF1 homologs from 13 species was constructed. As shown in Fig. 2b, the 21 TCF1 homologs can be divided into 3 clades. Clade I contains one *Arabidopsis* and four legume dicot species, Clade II species belong to non-legume dicotyledonous plants, and the remaining TCF1 homologs from monocots (*Zea mays*, *Oryza sativa*, *B. distachyon* and *Sorghum bicolor)* were grouped in clade-III (Fig. 2b). AtTCF1 was grouped with GmTCF1a and GmTCF1b in the clade I, indicating that *AtTCF1* has remarkably high homology with genes in legume plants and low homology with monocot genes (Fig. 2b). Taken together, GmTCF1a and GmTCF1b are more closely related to AtTCF1 than to monocots, which may be partly due to the fact that both soybean and *Arabidopsis* are dicotyledonous plants.

### Considering *GmTCF1a* as a putative orthologous gene of *AtTCF1*

**Fig. 3:**
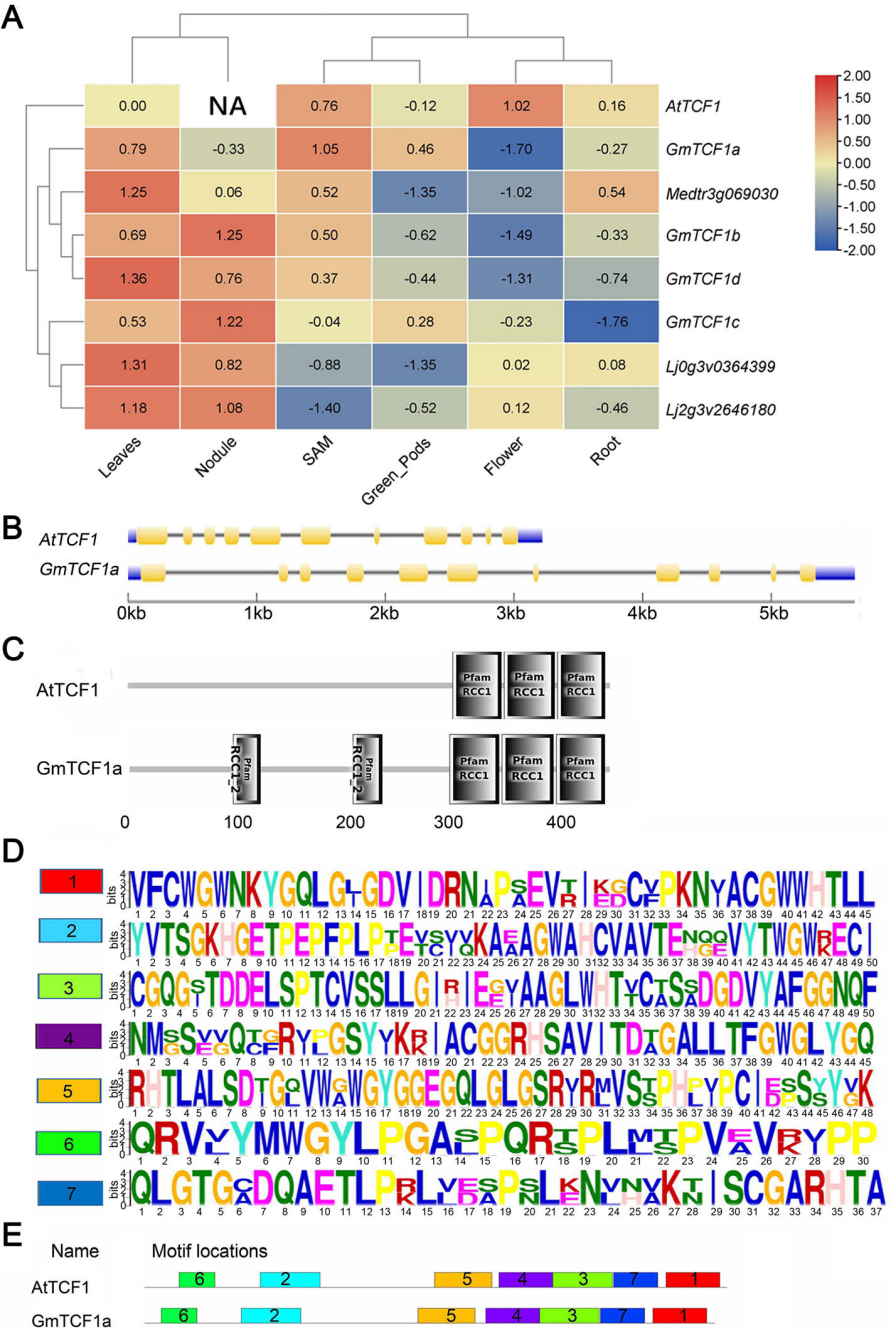
Expression pattern, domain and motifs analysis of AtTCF1 homologs. **A** Expression profiles of *AtTCF1* homologs from leguminous plants. SAM, shoot apical meristem; NA, no data for *AtTCF1* in nodule. **B** The intron–exon structure of *GmTCF1a* and *AtTCF1* genes. Introns and exons are represented by gray lines and yellow boxes, respectively. **C** Domain analysis of GmTCF1a and AtTCF1. The gray boxes show the RCC1 domain. **D** Conserved motifs of GmTCF1a and AtTCF1. Both GmTCF1 and AtTCF1 contain seven motifs indicated by different colors. **E** Distribution of conserved motifs along with the protein sequence. The motifs, numbered 1–7, are shown in different colored boxes. GmTCF1 and AtTCF1 share all the seven motifs

To explore the organ-specific expression pattern of these *GmTCF1s*, we retrieved the RNA sequencing expression data of *TCF1* homologs from public data (http://bar.utoronto.ca/) and arranged for heatmap production. As shown in Fig. 3a, *AtTCF1* was strongly expressed, while *GmTCF1s* were lowly expressed in flowers. The *GmTCF1a* gene was mainly expressed in the aerial parts of the plant (leaves, shoot apical meristem and pods) and had lower expression in flowers, nodules and roots. The expression of the remaining homologs (*GmTCF1b*, *GmTCF1c* and *GmTCF1d*) was also high in nodules compared to *GmTCF1a* (Fig. 3a), suggesting that *GmTCF1b*, *GmTCF1c* and *GmTCF1d* may acquire new function(s) in the underground parts of soybean. Further correlation analysis found that *AtTCF1* had a low correlation with the expression pattern of *GmTCF1a*, while *AtTCF1* had a strong negative correlation with the rest genes except *GmTCF1a* (Fig. S2). From the phylogenetic tree, *GmTCF1a* and *GmTCF1b* are the most homologous genes of *AtTCF1* (Fig. 2b). However, we found a low temperature-related cis-acting element [37] existing in the *GmTCF1a* promoter, but not in the *GmTCF1b* promoter (Fig. S3). Thus, *GmTCF1a* is considered as a putative orthologous gene of *AtTCF1* for further study*.*

The *GmTCF1a* gene has a gene structure similar to that of *AtTCF1*. Both genes contain 11 exons and 10 introns, but the *GmTCF1a* gene is much larger than *AtTCF1* because most of the introns in *GmTCF1a* are much longer than those in *AtTCF1* (Fig. 3b). *GmTCF1a* encodes a predicted protein of 477 amino acids with a calculated MW (molecular weight) of 50.199 kDa and a PI (protein isoelectric point) of 6.01. Interestingly, GmTCF1a contains 5 RCC1-like domains, two more than AtTCF1 (Fig. 3c), and shares 65% identity with AtTCF1 in its amino acid sequence (Fig. S4). The protein sequences of GmTCF1a and AtTCF1 were then subjected to MEME online analysis to find the common motifs. Seven conserved motifs were found in GmTCF1a and AtTCF1, which are consistent with the distribution of the motifs along with protein sequences (Fig. 3d and e). The conserved motifs indicate that GmTCF1a and AtTCF1 might exert functionally identical effects.

### Specific response of *GmTCF1a* gene to cold

**Fig. 4:**
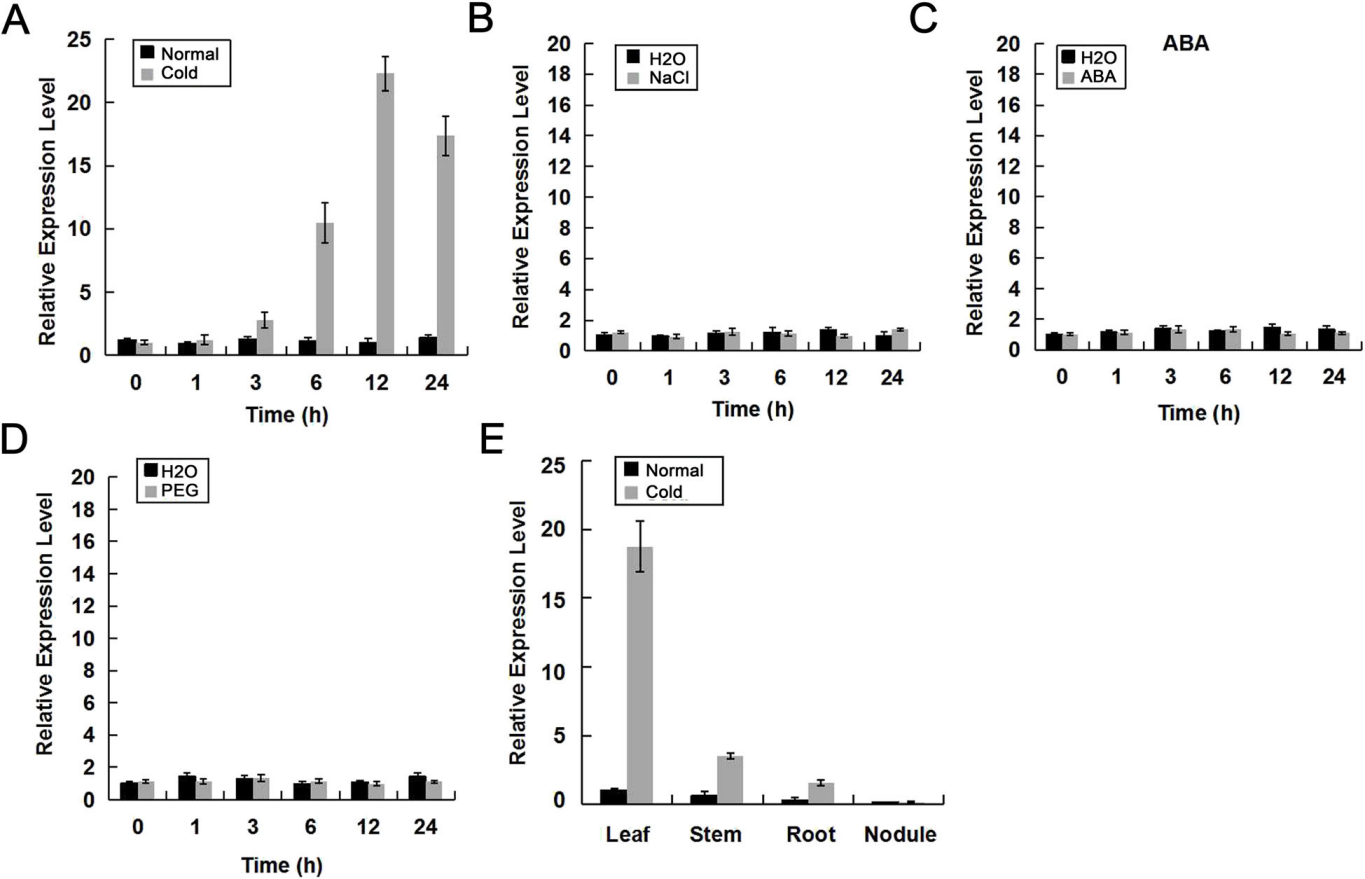
The relative expression level of *GmTCF1a* in soybean as determined by qRT-PCR. **A-D** Relative expression of *GmTCF1a* in response to cold (4 °C), ABA (100 μM ABA), PEG (15% PEG8000) and NaCl (200 mM NaCl) treatment at 0, 1, 3, 6, 12, and 24 h, respectively. **E** Expression of *GmTCF1a* in leaves, stems, roots and nodules with (Cold) or without (Normal) cold treatment at 4 °C for 12 h. The soybean *18S rRNA* gene was used as a reference gene. These experiments were repeated three times and error bars represent the standard deviation between three biological replicates

The expression of *GmTCF1a* gene in response to common adverse environmental conditions, such as cold, high soil salinity, drought and ABA was investigated by quantitative real-time PCR (qRT-PCR) [36]. Total RNA was extracted from soybean leaves treated with 4 °C, 200 mM NaCl, 15% PEG and 100 μM ABA at different time points. As shown in Figs. 4a and S5a, *GmTCF1a* had a very low expression level under normal conditions. Upon cold treatment, *GmTCF1a* expression was dramatically induced at 6 h and reached the highest level at 12 h (Figs. 4a and S5a). However, the expression of *GmTCF1a* did not change under NaCl, ABA and PEG treatments (Figs. 4b, c, d and S5b, c, d). In addition, the organ-specific expression pattern of *GmTCF1a* was examined using total RNA isolated from leaves, stems, roots and nodules of soybean plants with and without cold treatment. *GmTCF1a* was expressed at low levels in all organs tested under normal conditions; in sharp contrast, *GmTCF1a* was induced in these organs by cold treatment with the exception of the nodule and showed the highest expression level in the leaf (Figs. 4e and S5e). Taken together, these results suggest that *GmTCF1a* is a cold-responsive gene that may mediate plant responses to cold stress.

### Preferential expression of *GmTCF1a* in vascular tissues

**Fig. 5:**
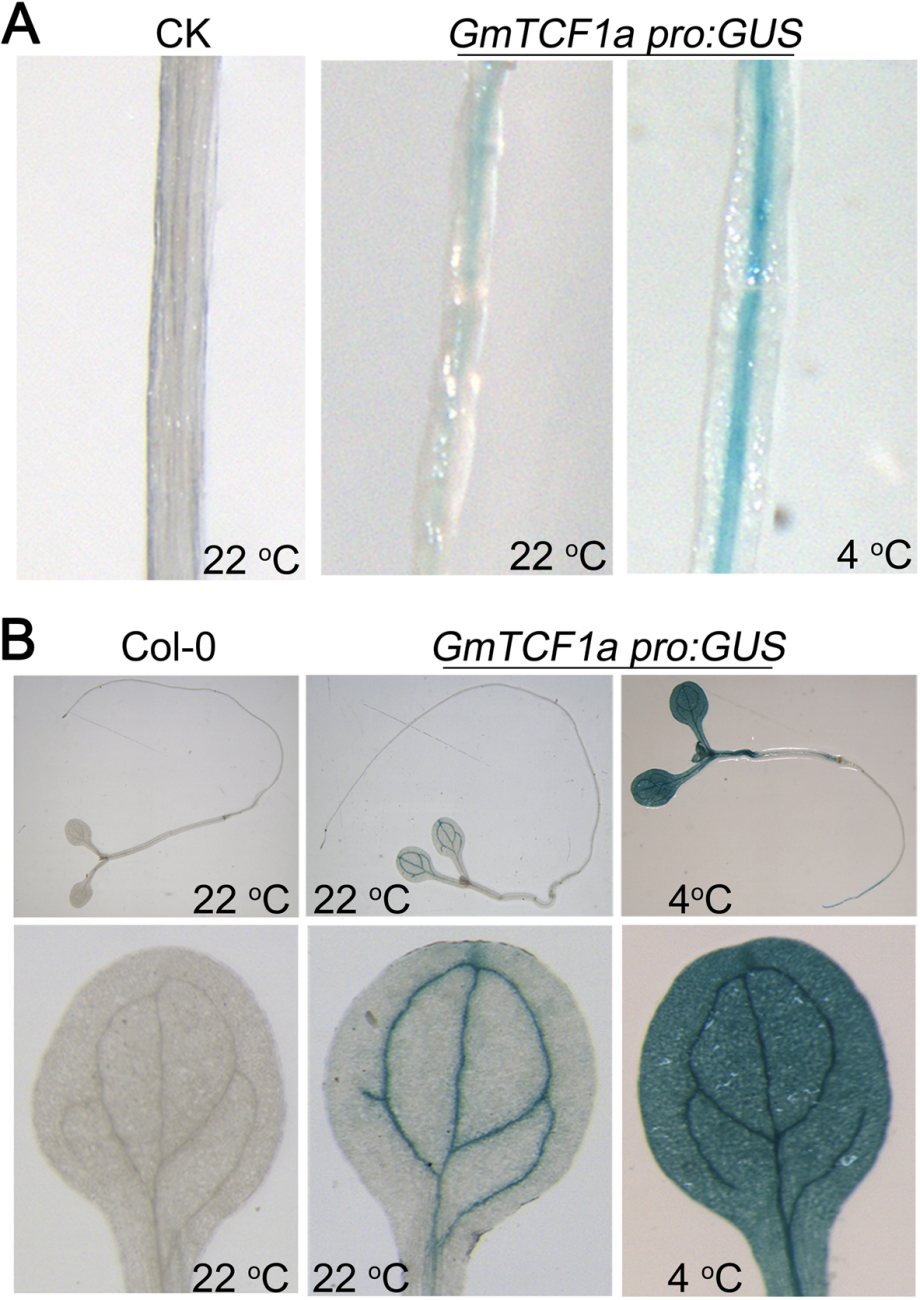
Histochemical staining of transgenic plants expressing *GmTCF1apro:GUS*. **A** GUS staining of soybean hairy roots expressing *GmTCF1apro:GUS* fusion with and without cold treatment. CK, Soybean hairy roots without *GmTCF1apro:GUS* fusion; **B** GUS staining of transgenic *Arabidopsis* seedlings containing *GmTCF1apro:GUS* fusion with and without cold treatment. Wild-type *Arabidopsis* Col-0 is defined as a control

To further examine the cell/tissue-specific expression of *GmTCF1a,* we constructed a binary vector containing a 2.0 kb *GmTCF1a* promoter that drives the *β-glucuronidase* (*GUS*) gene and then transformed *GmTCF1apro:GUS* into soybean root hairs and *Arabidopsis*. GUS histochemical staining revealed that *GmTCF1a* was expressed mainly in the root stele of soybean-transformed hairy roots, and its expression became much higher after cold treatment (Fig. 5a). In *Arabidopsis* plants expressing *GmTCF1apro:GUS*, GUS staining was detected only in the veins of cotyledons under normal conditions, whereas very strong GUS staining was observed in the entire cotyledons and hypocotyls after exposure to cold stress (Fig. 5b). These results indicate that *GmTCF1a* is highly induced by cold in both shoots and roots.

### GmTCF1a as a nuclear protein

**Fig. 6:**
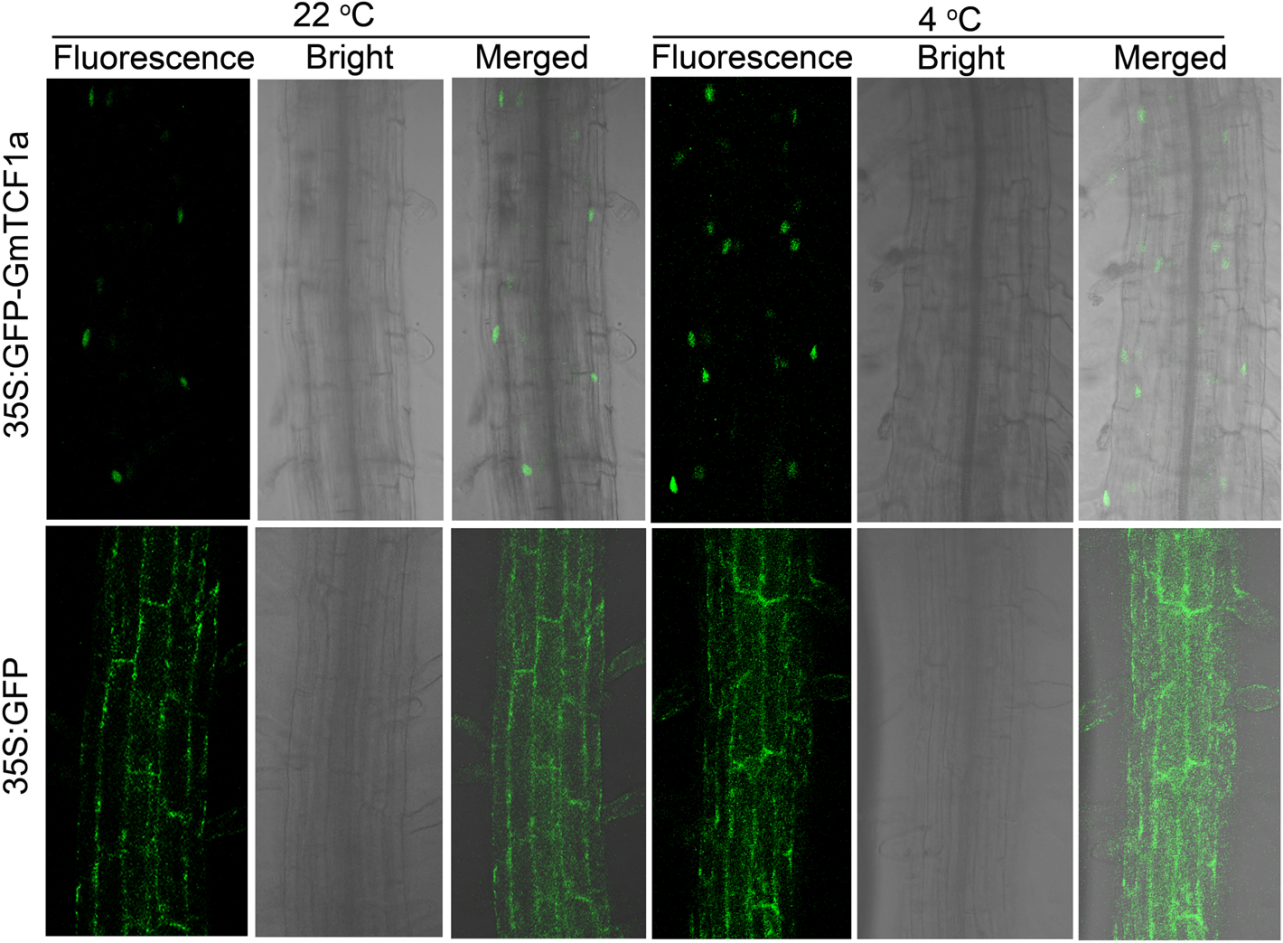
GmTCF1a localizes in the nucleus. Seven-day-old transgenic *Arabidopsis* seedlings expressing GFP-GmTCF1a and GFP were treated at 22 °C and 4 °C for 12 h before imaging analysis. The root cells in the elongation zone were observed using a 488 nm laser confocal microscope. Fluorescence, GFP channel; Bright, bright field; Merged, combination of GFP channel and bright field channel

To examine the subcellular localization, we made a construct harboring a *GFP-GmTCF1a* fusion gene under the control of the CaMV 35S promoter and generated transgenic *Arabidopsis* plants*.* Seven-day-old transgenic *Arabidopsis* seedlings were treated at 22 °C and 4 °C for 12 h, respectively. Confocal microscopy revealed that the GFP-GmTCF1a fusion proteins were localized in the nucleus under normal condition, contrasting sharply with the ubiquitous localization of GFP in the entire root cell (Fig. 6). Cold stress did not alter nuclear localization of GmTCF1a (Fig. 6). Thus, GmTCF1a and AtTCF1 display the same subcellular localization, and play roles in the nucleus during cold stress [36].

### Enhancement of freezing tolerance in *Arabidopsis* plants by ectopic expression of *GmTCF1a*

**Fig. 7:**
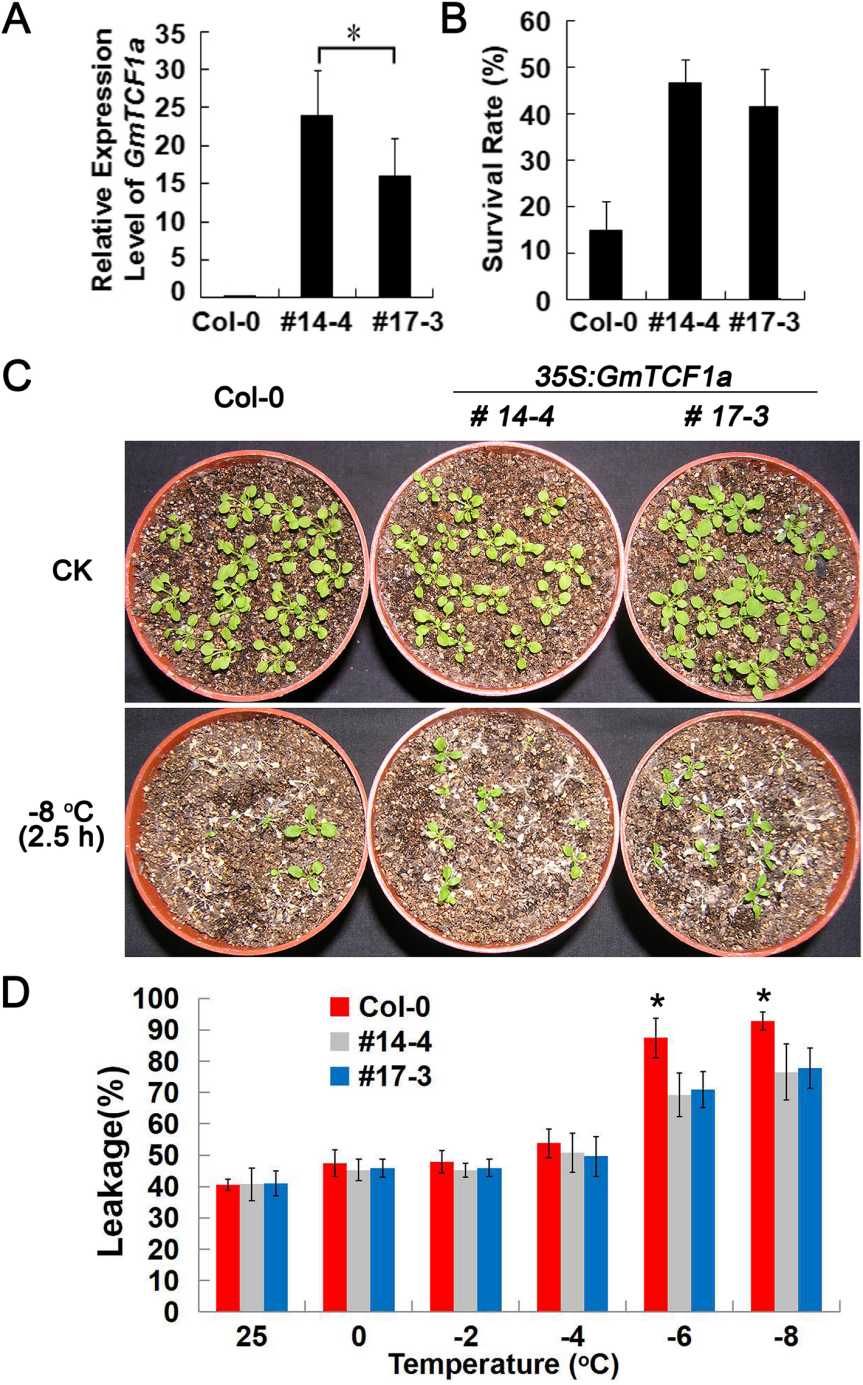
Increase in cold tolerance by overexpression of *GmTCF1a* in *Arabidopsis*. **A** The expression level of *GmTCF1a* in two *35S:GmTCF1a* transgenic plants. The *UBC* gene was used as a control. B The survival rate of wild-type and *35S:GmTCF1a* lines after freezing treatment from b. **C** Freezing assay of wild-type and two transgenic *Arabidopsis* lines (#14–4 and #17–3) at − 8 °C for 2.5 h after cold acclimation. Photographs were taken 7 days after freezing treatment. **D** Electrolyte leakage from wild-type and two *35S:GmTCF1a* lines after being treated at temperatures of 0, − 2, − 4, − 6, and − 8 °C for 0.5 h. Error bars represent the Mean ± SD from three technical replicates. One-way ANOVA with a Tukey test was performed to explore the significant differences at − 6 and − 8 °C treatment, and * indicates *P* < 0.05. These experiments were repeated three times and obtained the same results

To investigate whether *GmTCF1a* acts as a key regulator of the response of plants to cold stress, we constructed a binary vector harboring *35S:GmTCF1a* and transformed it into *Arabidopsis* plants. After three generations of selection, we obtained two homozygous transgenic lines that constitutively overexpress *GmTCF1a* (Fig. 7a). Three-week-old transgenic plants and wild-type plants were treated at 4 °C for 7 days before being subjected to − 8 °C for 2.5 h. Survival rates were calculated after 7 days of recovery under normal growth conditions. The survival rates of the two representative *35S:GmTCF1a* transgenic *Arabidopsis* lines from independent transgenic events were about 47% (line #14–4) and 42% (line #17–3), respectively; by contrast, the survival rate of wild-type plants was only 15% (Fig. 7b and c). The result indicates that overexpression of *GmTCF1a* greatly elevates the tolerance of the transgenic plants to low temperature.

Cold stress can destroy the plant cell walls and cause the cytosol to leak out. Therefore, the lower rate of electrolyte leakage reflects higher cold tolerance. An electrolyte leakage assay was performed to compare the freezing tolerance between *GmTCF1a* overexpression plants (#14–4 and #17–3) and wild-type plants. As shown in Fig. 7d, there were no significant difference in electrolyte leakage rates between transgenic plants and wild-type plants when treated at 25, 0, − 2 and − 4 °C for 0.5 h; however, the percentages of electrolyte leakage were significantly lower in *GmTCF1a* overexpression plants than in wild-type plants when the temperature was reduced to − 6 and − 8 °C (Fig. 7d). Although the electrolyte leakage of #17–3 were slightly higher than #14–4, but no significant differences were found. Taken together, the result indicates that the overexpression of *GmTCF1a* can improve the freezing tolerance of *Arabidopsis* plants.

We also tested the role of overexpression *AtTCF1* in *Arabidopsis*. As shown in Fig. S6, the survival rates of two independent homozygous *35S:AtTCF1* transgenic *Arabidopsis* lines were about 37% (line #3–6) and 48% (line #12–2), respectively; by contrast, the survival rate of wild-type plants was only 19%. The result indicates that overexpression of *AtTCF1* greatly elevates the tolerance of the transgenic plants to low temperature. The electrolyte leakage assay also demonstrated that *AtTCF1* overexpression plants reduced the percentage of leakage at − 8 °C (Fig. S6d). In summary, these results indicate that overexpression of *AtTCF1* or *GmTCF1a* can improve the freezing tolerance of *Arabidopsis* plants.

### Upregulation of COR15a by *GmTCF1a* overexpression in *Arabidopsis* plants

**Fig. 8:**
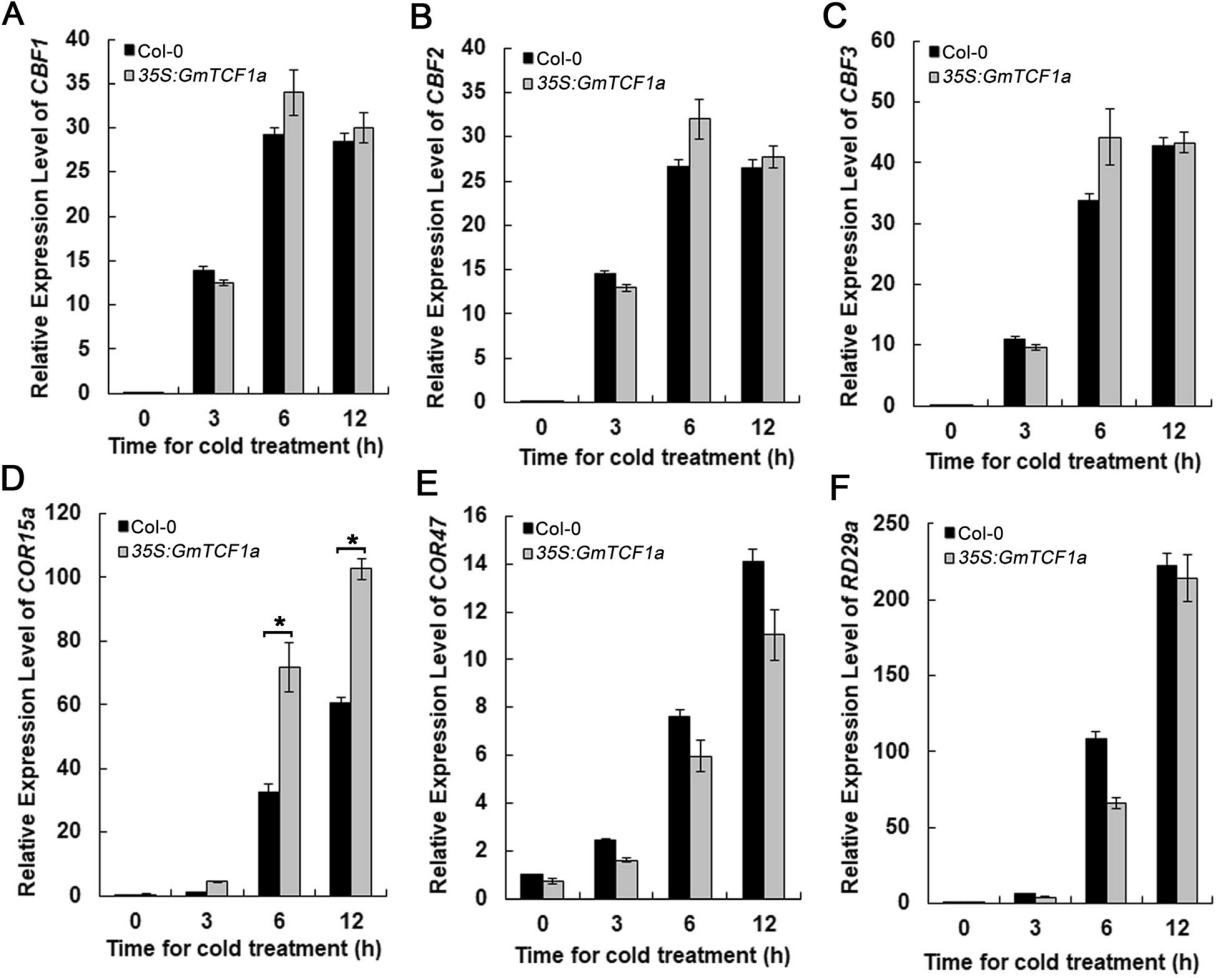
The relative expression level of cold-responsive genes in *35S:GmTCF1a* lines. **A-F** Relative expression levels of *AtCBF1, AtCBF2, AtCBF3, AtCOR15a, AtCOR47* and *AtRD29a* in wild-type and *35S:GmTCF1a* transgenic *Arabidopsis* plants (#17–3). Three-week-old plants were subjected to a low temperature (4 °C) and samples were harvested at the indicated time points. These experiments were repeated three times and error bars represent the standard deviation between three biological replicates. *, *t*-test (*P* < 0.05)

To determine whether overexpression of *GmTCF1a* affects cold-responsive gene expression in *Arabidopsis*, total RNA was extracted from cold-treated transgenic and wild-type *Arabidopsis* seedlings. Due to the same freezing tolerance phenotype of line #17–3 and #14–2, we selected line #17–3 as a representative transgenic plants for gene expression analysis. Six cold responsive genes *CBF1*, *CBF2*, *CBF3*, *COR15a*, *COR47* and *RD29a* were selected [21, 24, 30] and examined by qRT-PCR. As a result, the expression of *CBF1*, *CBF2*, *CBF3*, *COR47* and *RD29a* was not altered in the line #17–3 compared to wild-type plants. However, the expression of *COR15a* was much higher in transgenic plants than in wild-type plants at the tested time points (Fig. 8). This result suggests that *GmTCF1a* enhances the cold tolerance of plants by up-regulating *COR15a* expression. We also investigated the level of *COR15a* in *AtTCF1* overexpression transgenic lines (#12–2), and *COR15a* was also higher in transgenic plants than in wild-type plants (Fig. S7). In summary, the increased cold tolerance in overexpressed *AtTCF1* or *GmTCF1a* plants is associated with the up-regulated levels of *COR15a*.

## Discussion

Low temperature is one of the primary abiotic stresses, which negatively affects the growth and productivity of soybean. Identifying the determinants of soybean tolerance to low temperature is crucial for the genetic improvement of soybean stress tolerance. In this study, we identified *GmTCF1a* in soybean as a putative orthologous gene of *Arabidopsis TCF1*, which is specifically induced by cold stress and regulates freezing tolerance in *Arabidopsis*. These findings reveal a conserved mechanism by which plants respond to low freezing temperatures and provide novel insights into the genetic improvement of freezing tolerance in soybean.

### *GmTCF1a* as a putative orthologous gene of *AtTCF1*

Several RCC1 family proteins, such as UVR8, TCF1, RUG3 and SAB1, have been characterized and exhibit different biological functions in response to abiotic stresses [36, 38–43]. In *Arabidopsis*, AtTCF1 regulates plant tolerance to freezing stress by reprogramming cell wall properties [36]. Like *Arabidopsis*, soybean is also a temperate plant and is susceptible to low temperatures. We hypothesized that soybean might adopt a similar mechanism to cope with low temperature stress. Indeed, we found four homologous genes of *AtTCF1* in soybean. Apparently, *GmTCF1s* exhibit evolutionary diversity in soybean. GmTCF1s contain several RCC1-like domains (Fig. S8) and share a high degree of sequence identity and similarity (Fig. S4). It is well known that whole-genome duplication (WGD) plays a central role in the expansion of the soybean gene family [44], with two independent duplications occurring approximately 59 and 13 million years ago, resulting in 75% of genes in multiple copies in soybean [45]. Therefore, four *GmTCF1s* can be evolved through WGD. Among the four *GmTCF1s*, *GmTCF1a* is considered a putative orthologous gene of *AtTCF1*. Firstly, phylogenetic analysis showed that *GmTCF1a* was the closest homolog of *AtTCF1* (Fig. 2b). Secondly, a low-temperature-related cis-acting element was present in the *GmTCF1a* promoter (Fig. S3). Finally, *GmTCF1a* was barely expressed in legume-specific organ-nodules (Fig. 3a). Thus, the *GmTCF1a* gene has a high degree of evolutional conservation in soybean. In addition, *GmTCF1a* has longer introns than *AtTCF1* in their genomic sequence, and three putative splicing variants of *GmTCF1a* from the phytozome database were found, whether these variants are responsive to cold stress or cold tolerance need to be further investigated.

### *GmTCF1a* as a functional ortholog of *AtTCF1*

Indeed, our further experiments demonstrated that *GmTCF1a* is a functional ortholog of *TCF1*. First, *GmTCF1a* and *AtTCF1* are specifically responsive to cold temperature [36]. Under normal conditions, both *GmTCF1a* and *AtTCF1* were expressed at very low levels in leaves, stems and roots. After 12 h of cold stress, the expression of *GmTCF1a* and *AtTCF1* was strongly induced in leaves, stems and roots (Figs. 4e and 5) [36]. Most importantly, the expression of both genes was specifically and highly induced by cold stress, but not by ABA, PEG and salt stress (Fig. 4a,b,c,d) [36]. In transgenic soybean roots, despite the fact that a 6 h GUS response at 37 °C might reverse the cold-induced upregulation of *GmTCF1apro:GUS* expression in certain cells, *GmTCF1a* was highly induced in vascular tissue by cold stress, while *GmTCF1a* transcription was predominantly induced on the aboveground of *Arabidopsis* (Fig. 5). Second, GmTCF1a and AtTCF1 display the same subcellular localization, and they are both localized in the nucleus (Fig. 6). Third, both *GmTCF1a* and *AtTCF1* mediate the response of *Arabidopsis* to freezing temperature. We showed that ecotopic *GmTCF1a* or *AtTCF1* enhanced plant tolerance to freezing temperatures (Figs. 7 and S6). Finally, like AtTCF1, GmTCF1a involved in freezing tolerance is independent of CBFs [36], because ecotopic expression of *GmTCF1a* does not alter the expression of *CBFs* in *Arabidopsis.* These results demonstrate that *GmTCF1a* is indeed a functional ortholog of *AtTCF1* and that soybean may also have a conserved TCF1-mediated mechanism in response to freezing and cold.

### *GmTCF1a* as a positive regulator of freezing tolerance

In *Arabidopsis*, we showed that the loss of *AtTCF1* function exhibited enhanced tolerance to freezing temperatures [36]. In this study, we showed that overexpression of *AtTCF1* also dramatically enhanced freezing tolerance in transgenic *Arabidopsis* (Fig. S6), suggesting a complex mechanism for AtTCF1 in the cold signaling pathway. However, we found that constitutive expression of either *GmTCF1a* or *AtTCF1* elevated the level of *COR15a*. *COR15a* encodes a chloroplast-targeting polypeptide, and constitutive expression of *COR15a* enhances its in vivo freezing tolerance to chloroplasts under cold acclimation or nonacclimated treatment in *Arabidopsis* [46, 47]. Thus, it is possible that enhancing freezing tolerance of chloroplasts may lead to freezing tolerance in transgenic plants. The manner in which GmTCF1a regulates *COR15a* levels remains unclear. RCC1-like protein usually associates with downstream genes and regulates their expression by affecting histone modifications in the gene promoter region [36, 41, 48, 49], and we speculate that GmTCF1a may influence histone modifications in the *COR15a* promoter or that GmTCF1a interacts with an unknown gene protein, which in turn regulates *COR15a* levels. In addition, it is known that strict control of lignin homeostasis in the cell wall is required for freezing tolerance [50]. Reduced lignin deposition within the cell wall in the *tcf1* mutant during cold stress may increase cell wall permeability and protect the cells from freezing damage [36]. Overexpression of *AtTCF1* is likely to also enhance plant stress tolerance and maintain the lignin content at an optimal level. We speculate that overexpression of *GmTCF1a* has a significant effect on influencing cellular lignin content. Further studies on lignin dynamics in response to freezing temperature and the role of GmTCF1a in this process will provide novel insights into the mechanisms of freezing tolerance in plants.

## Conclusions

In conclusion, this study reports a RCC1-like gene, *GmTCF1a*, which is a novel gene associated with cold acclimation in soybean. *GmTCF1a* is specifically induced by cold stress and enhances freezing tolerance to transgenic *Arabidopsis*. It is still necessary to investigate the exact molecular and physiological mechanisms by which *GmTCF1a* and its paralogs regulate freezing tolerance in soybean*.* The application of *GmTCF1a* in improving cold tolerance in soybean is also worth exploring.

## Methods

### Identification of *GmTCF1s* and structure analysis

The coding sequence (CDS) of AtTCF1 was retrieved from the TAIR website (https://www.arabidopsis.org/). To identify soybean RCC1-like proteins, the soybean and *A. thaliana* protein sequences and genome annotations were downloaded from the Phytozome database (https://phytozome.jgi.doe.gov/pz/portal.html). First, the HMM (Hidden Markov Model) of the RCC1 (PF00415 and PF13540) domain was obtained from the PFAM database (http://pfam.xfam.org/), and the soybean RCC1s (GmRCC1s) and *Arabidopsis* RCC1s (AtRCC1s) was predicted using the HMMER software. Second, the protein sequences were then used to construct HMMs [51]. Third, the proteins containing conserved RCC1 domains were considered as GmRCC1s and AtRCC1s. The RCC1 domains were verified by SMART software (http://smart.embl-heidelberg.de/) and the PFAM database. Finally, using AtRCC1s sequence as a query to search the soybean proteome (BLASTP), proteins that met the BLASTP threshold conditions (E-value ≤1e-5 and identity ≥50%) were retained as the final GmRCC1s and AtRCC1s family members. PI and MW were analyzed by the Compute PI/MW tool (http://expasy.org/tools/pi_tool.html). The gene structures of *GmTCF1a* and *AtTCF1* were drawn based on cDNA sequences using the online software GSDS 2.0 (http://gsds.gao-lab.org/) [52]. The promoter sequence of *GmTCF1s* with a length of 2000 bp upstream of the translation site (ATG) was obtained from Phytozome, and the *cis*-element was analyzed on the PlantCARE website (http://bioinformatics.psb.ugent.be/webtools/plantcare/html/). The RCC1 domains of GmTCF1a and AtTCF1 were predicted using SMART tools (http://smart.embl.de/smart/set_mode.cgi?NORMAL=1). The sequence alignment between GmTCF1a and AtTCF1 was performed using the CLUSTALW program (https://www.genome.jp/tools-bin/clustalw) and MEGA 7.0 software [53].

### Phylogenetic and bioinformatics analysis

The homologous protein sequences of GmTCF1a from *A. thaliana*, *Glycine Max*, *Oryza sativa*, *Medicago truncatula*, *Phaseolus vulgaris*, *Populus trichocarpa*, *Linum usitatissimum*, *Gossypium raimondii*, *Zea mays*, *B. distachyon*, *Sorghum bicolor* and *C. sativus* were retrieved from Phytozome using the BLASTP program, and the protein homologs of *Lotus japonicus* were retrieved from the Lotus base (https://lotus.au.dk/). The sequences were aligned by the CLUSTALW program and a phylogenetic tree was constructed using MEGA 7.0 software with the neighbor-joining method [53]. The collinearity analysis was performed by the MCScanX software (http://chibba.pgml.uga.edu/mcscan2/MCScanX.zip) and the figure was constructed by TBtools (https://github.com/CJ-Chen/TBtools), respectively. The analysis of conserved motifs for GmTCF1a and AtTCF1 was performed by MEME online tool with default parameters except for a limit of seven numbers of motifs (http://meme-suite.org/tools/meme) [54].

### Tissue expression pattern analysis

Tissue-specific expression data (Normalized FPKM/TMP) of eight *AtTCF1* homologs were downloaded from eFP browser (http://bar.utoronto.ca/) and *Lotus japonicus* Expression Atlas (https://lotus.au.dk/expat/). Three biological replicates of these data from a subset of the tissues were used in the heatmap. Heatmap was built using row as scale type, the rows and columns of the heatmap was clustered. The correlation of the expressed data were analyzed using TBtools (SupCorrPlot function).

### Growth of soybean and treatments

Soybean (*G. max* L.) Williams 82, a sequenced cultivar (originally from America), is a gift from the laboratory of Peter M. Gresshoff (University of Queensland, Australia). Williams 82 were grown on vermiculite under 16/8 h (light (6:00–22:00)/dark) photoperiod at 27 °C for 3 weeks and then subjected to various abiotic stresses. For cold treatment, the pots were exposed to 4 °C at 9:00 am, and the first trifoliolate leaves were harvested after 1, 3, 6, 12, and 24 h treatments, respectively. For abscisic acid (ABA), PEG8000 and salt treatments, the roots of the seedlings were pulled out from the vermiculite and dipped into the solutions of 100 μM ABA, 15% PEG8000 and 200 mM NaCl at 9:00 am, respectively, and then the first trifoliolate leaves were harvested after 1, 3, 6, 12, and 24 h treatments.

To identify the expression pattern of *GmTCF1a*, soybean seeds were surface sterilized with 70% ethanol and then imbibed at 28 °C for 3 days. The germinated seeds were then inoculated with *B. japonicum* USDA110 (OD = 0.08) for 30 min and then transferred to the long test tubes with a low-nutrient solution containing 0.03 g Ca(NO_3_)-4H_2_O, 0.1 g CaCl_2_-2H_2_O, 0.1 g KH_2_PO_4_, 0.15 g Na_2_HPO_4_-12H_2_O, 0.12 g MgSO_4_-7H_2_O, 0.05 g/L ferric citrate, plus 1 mL of micro-element H_2_BO_3_ 2.86 mg, MnSO_4_ 1.81 mg, ZnSO_4_ 0.22 mg, CuSO_4_ 0.8 mg, and H_2_MO_4_ 0.02 mg/L. The inoculated seedlings were grown at 27 °C under a photoperiod of 16/8 h (light (6:00–22:00)/dark) with a light intensity of 108.38 μmol/m^2^s. Whole plants were exposed to 4 °C at 9:00 am, then the first trifoliolate leaves, stems, roots and nodules were harvested at 21:00 pm, and samples were immediately frozen in liquid nitrogen and stored at − 80 °C for RNA isolation.

### RNA isolation and real-time quantitative PCR (qRT-PCR)

Total RNA was extracted using the PureLink Plant RNA Reagent (ThermoFisher Scientific, cat. #12322012). First-strand cDNA was synthesized using the M-MLV reverse transcriptase enzyme (Promega). qRT-PCR was performed on an ABI PRISM 7500 real-time PCR system. The procedure for real-time PCR is as follows. The PCR reaction solution (total 20 μL) containing 10 μL of SYBR Premix Ex Taq, 50 ng cDNAs, 0.2 μM of each primer, 0.4 μL of ROX Reference DyeII. The PCR mixtures were heated at 95 °C for 30 s, followed by 40 cycles of amplification (95 °C for 5 s, 60 °C for 34 s). The results were analyzed using 7500 system software with 2^-ΔΔCT^ method. The *18S rRNA* gene was used as a control. The sequences of the qRT-PCR primers are shown in Table S1.

### Analysis of *GmTCF1a pro:GUS* expression pattern

The fragment covering 2000 bp immediately upstream of the *GmTCF1a* coding region was amplified with the primers listed in Table S1. The promoter was cloned into the *Pst*I and *Bam*HI sites preceding a GUS gene in the pCAMBIA1391 vector. The destination vector was transferred into *A. tumefaciens* strain GV3101. Floral-dip method was used for stable transformation in *Arabidopsis* [55]. Transgenic plants were screened on MS medium containing 20 mg/L hygromycin B. After three generations of screening, we obtained two independent homozygous transgenic plants. The transformation of soybean hairy roots was performed based on a small amount of modification of soybean by *Agrobacterium rizogenes*-mediated methods [56]. GUS reactions of transgenic materials with and without cold treatment (4 °C, 12 h) were performed for 6 h at 37 °C in the dark. Chlorophyll was removed by washing several times with 70% ethanol and then representative images were photographed.

### GFP-GmTCF1a subcellular localization assay

The coding sequence of *GmTCF1a* was amplified from cDNA with the primers shown in Table S1. The sequence was cloned into the pEZR(K)-LC vector between the *Kpn*I and *Bam*HI sites after a GFP gene. This construct was then transformed into Col-0 (ordered from the *Arabidopsis* Biological Resource Center) via *Agrobacterium*-mediated transformation [55]. Homozygous transgenic plants were obtained after three generations of selection and subcellular localization was observed using a confocal microscope with a 488 nm laser (Leica SP8).

### Overexpression of *GmTCF1a* in *Arabidopsis*

The *GmTCF1a* CDS was cloned into the *Xba*I and *Kpn*I sites of the pCAMBIA1300 vector. The recombinant vector was transformed into Col-0 via *Agrobacterium*-mediated transformation [55]. T_0_ generation seeds were screened with Kanamycin (75 mg/mL). Homozygous transgenic plants were obtained after three generations of Kanamycin screening. Two homozygous transgenic lines were obtained with high *GmTCF1a* levels.

### Freezing tolerance assay for the *GmTCF1a* overexpression plants

*A. thaliana* seeds were sterilized by soaking in 50% bleach for 5 min, after which they were washed five times with sterile distilled water and then placed onto solidified MS medium containing 2% sucrose with the pH adjusted to 5.7. The 7-day-old transgenic and wild-type seedlings were transferred to pots for another 2 weeks growth (21–23 °C chamber), and then treated at 4 °C for 7 days before subjected to − 8 °C for 2.5 h. The survival percentage of plants (number of alive plants/total number of plants) were calculated after 7 days of recovery (16: 8 h light-dark period at 22 °C).

### Electrolyte leakage measurement

Electrolyte leakage assays were performed with minor modifications as described by Ristic and Ashworth [57]. Briefly, three excised leaflets were placed in a 15 mL plastic tube containing 100 μL dH_2_O, and then incubated in a freezing bath with 0 °C. The temperature of bath was programmed to drop to − 8 °C in a 2 °C reduction over 30 min. The tubes were taken out from the bath and immediately placed on ice when the designated temperature was reached. The leaflets in 15 mL tubes were then transferred to 50 mL tubes containing 25 mL of dH_2_O, the conductivity of the solution (E1) in 50 mL tubes was measured after shaking. The solutions’ conductivities (E2) were measured again after the leaflets were autoclaved. The electrolyte leakage was calculated as the percentage of E1/E2 [27].

### Expression of cold responsive genes in *GmTCF1a* overexpression transgenic *Arabidopsis*

Three-week-old transgenic plants were treated at 4 °C and the leaves were harvested at 0, 3, 6 and 12 h, respectively. RNA extraction and cDNA synthesis were performed as mentioned above. The specific primers for cold-responsive genes were listed in Table S1.

## Supplementary Information

**Additional file 1: Fig. S1.** Collinear genes of *AtTCF1* in soybean.

**Additional file 2: Fig. S2**. Correlation of eight *AtTCF1* homologs based on their expression levels.

**Additional file 3: Fig. S3**. *cis*-elements in the promoter region of *AtTCF1* and *GmTCF1s.*

**Additional file 4: Fig. S4.** Protein sequence alignment of AtTCF1 and GmTCF1s.

**Additional file 5: Fig. S5.** Expression pattern of *GmTCF1a*.

**Additional file 6: Fig. S6.** Overexpression of *AtTCF1* in *Arabidopsis* increases plant cold tolerance.

**Additional file 7: Fig. S7.** The relative expression level of *AtCOR15a* in wild-type and *35S:AtTCF1a* transgenic *Arabidopsis* plants.

**Additional file 8: Fig. S8.** Domain analysis of GmTCF1s.

**Additional file 9: Table S1.** Primers used in this study.

## Data Availability

All data generated or analysed during the current study are available in this article and its supplementary information files. The accession numbers of AtTCF1 (At3G55580) homologs are as follows: *G. max*, GmTCF1a (Glyma.02G250700); GmTCF1b (Glyma.14G066000); GmTCF1c (Glyma.11G223000); GmTCF1d (Glyma.18G034600); *Oryza sativa*, LOC Os05g38270 and LOC Os01g62810; *Medicago truncatula*, Medtr3g069030; *Phaseolus vulgaris*, Phvul.008G230700 and Phvul.001G236500; *Populus trichocarpa*, Potri.008G059800 and Potri.010G199100; *Linum usitatissimum*, Lus10004700.g and Lus10040266.g; *Gossypium raimondii*, Gorai.011G268700 and Gorai.012G072600; *Zea mays*, GRMZM2G337819 and GRMZM2G302245; *B. distachyon*, Bradi2g54850; *Sorghum bicolor*, Sobic.003G354900 and *C. sativus*, Cucsa.165020.

## Abbreviations

TCF1: Tolerant to Chilling and Freezing 1
RCC1: Regulator of Chromosome Condensation Protein 1
CBF: C-repeat binding transcription factor
CRPK1: Cold-Responsive Protein Kinase 1
ICE1: Inducer of CBF Expression 1
bHLH: A basic Helix-Loop-Helix
COR: Cold Regulated genes
DRE: Dehydration-responsive-element
CRT: C-repeat
BCB: Blue-Copper-Binding
