# Supplementary material for: Enhancement of plant cold tolerance by soybean RCC1 family gene GmTCF1a
Source: BMC Plant Biol. 2021 Aug 12;21:369. doi: 10.1186/s12870-021-03157-5 (PMC8359048; doi:10.1186/s12870-021-03157-5)
Supplement: Supplementary file 1 — Additional file 1: Fig. S1. Collinear genes of AtTCF1 in soybean. [file 12870_2021_3157_MOESM1_ESM.pdf]

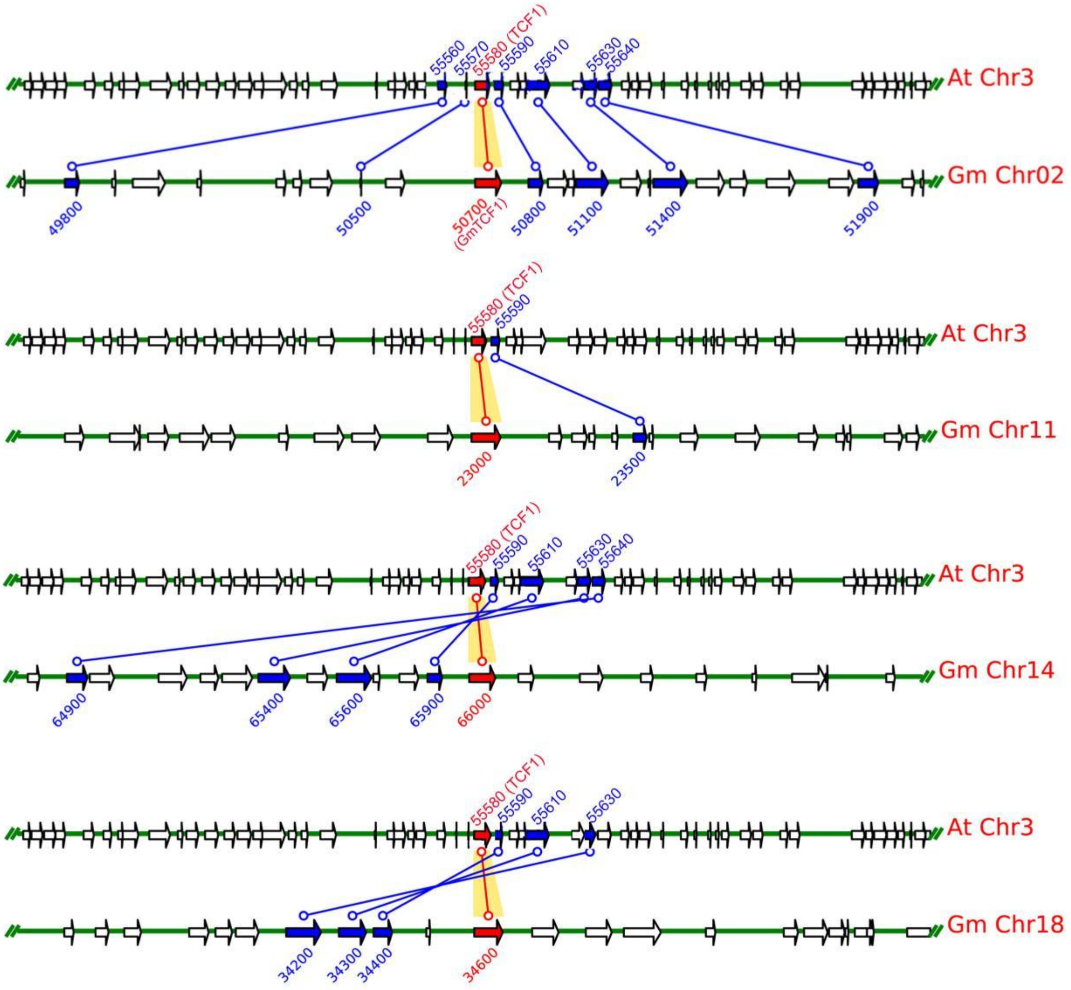

Additional file 1: Figure S1. Collinear genes of *AtTCF1* in soybean. The data is from plant genome duplication website (http://chibba.agtec.uga.edu/duplication/index/locus). Red and blue arrows show that *AtTCF1* is collinear with four homologous genes in soybean.
