# Supplementary material for: Enhancement of plant cold tolerance by soybean RCC1 family gene GmTCF1a
Source: BMC Plant Biol. 2021 Aug 12;21:369. doi: 10.1186/s12870-021-03157-5 (PMC8359048; doi:10.1186/s12870-021-03157-5)
Supplement: Supplementary file 2 — Additional file 2: Fig. S2. Correlation of eight AtTCF1 homologs based on their expression levels. [file 12870_2021_3157_MOESM2_ESM.pdf]

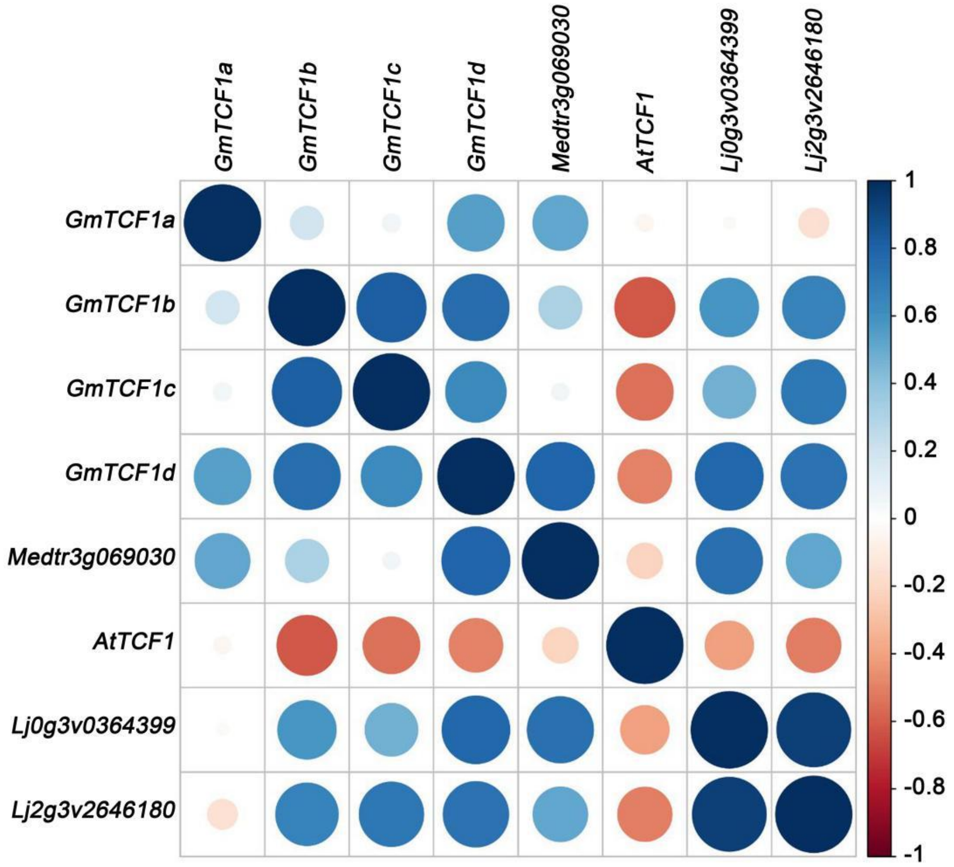

Additional file 2: Figure S2. Correlation of eight *AtTCF1* homologs based on their expression levels. Darkblue colour represents a strong positive correlation, white colour represents a poor correlation and darkred colour represents a strong negative correlation.
