# Supplementary material for: Enhancement of plant cold tolerance by soybean RCC1 family gene GmTCF1a
Source: BMC Plant Biol. 2021 Aug 12;21:369. doi: 10.1186/s12870-021-03157-5 (PMC8359048; doi:10.1186/s12870-021-03157-5)
Supplement: Supplementary file 3 — Additional file 3: Fig. S3. cis-elements in the promoter region of AtTCF1 and GmTCF1s. [file 12870_2021_3157_MOESM3_ESM.pdf]

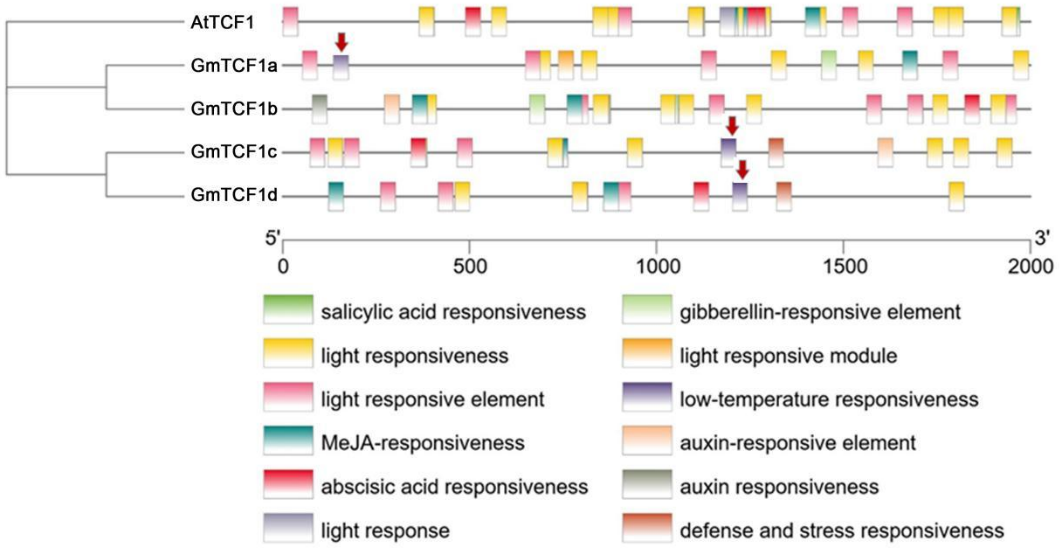

Additional file 3: Figure S3. *cis*-elements in the promoter region of *AtTCF1* and *GmTCF1s*. The 2000 bp promoters region of five genes (*AtTCF1*, *GmTCF1s*) were used for *cis*-elements analysis. The red arrows show the *cis*-acting elements involved in low-temperature responsiveness.
