# Supplementary material for: Enhancement of plant cold tolerance by soybean RCC1 family gene GmTCF1a
Source: BMC Plant Biol. 2021 Aug 12;21:369. doi: 10.1186/s12870-021-03157-5 (PMC8359048; doi:10.1186/s12870-021-03157-5)
Supplement: Supplementary file 4 — Additional file 4: Fig. S4. Protein sequence alignment of AtTCF1 and GmTCF1s. [file 12870_2021_3157_MOESM4_ESM.pdf]

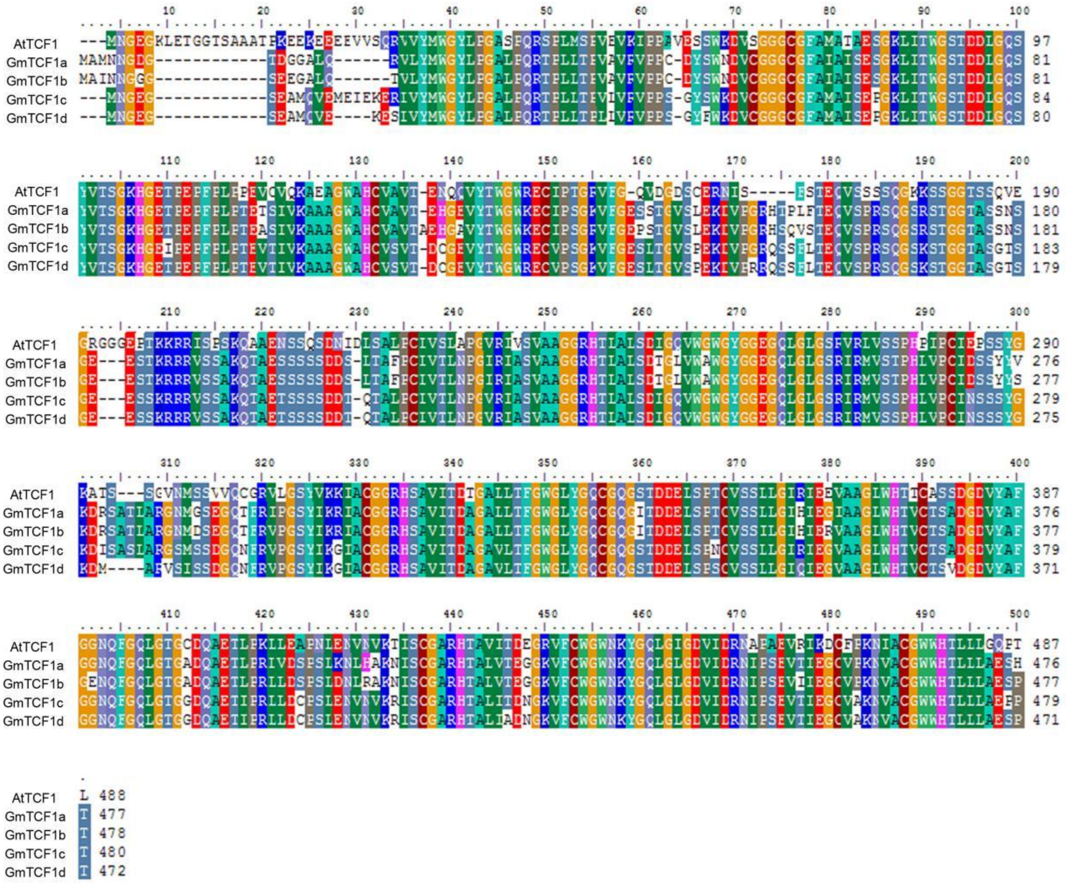

Additional file 4: Figure S4. Protein sequence alignment between AtTCF1 and GmTCF1s. The sequence alignment was performed using the CLUSTALW program and MEGA 7.0 software.
