# Supplementary material for: Enhancement of plant cold tolerance by soybean RCC1 family gene GmTCF1a
Source: BMC Plant Biol. 2021 Aug 12;21:369. doi: 10.1186/s12870-021-03157-5 (PMC8359048; doi:10.1186/s12870-021-03157-5)
Supplement: Supplementary file 5 — Additional file 5: Fig. S5. Expression pattern of GmTCF1a. [file 12870_2021_3157_MOESM5_ESM.pdf]

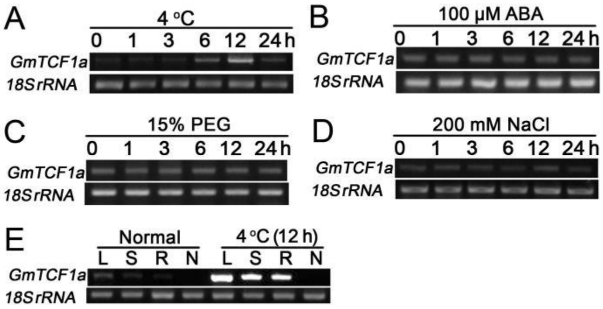

Additional file 5: Figure S5. Expression pattern of *GmTCF1a*. (A-D) Expression of *GmTCF1a* in response to cold, ABA, PEG and NaCl treatment. Three-week-old seedlings were treated with 4 ℃, 100 μM ABA, 15% PEG8000 and 200 mM NaCl, respectively. (E) Transcriptional level of *GmTCF1a* in different soybean organs with and without cold treatment. L, leaves; S, stems; R, roots and N, nodules. The soybean *18S rRNA* gene was used as an internal control.
