# Supplementary material for: Enhancement of plant cold tolerance by soybean RCC1 family gene GmTCF1a
Source: BMC Plant Biol. 2021 Aug 12;21:369. doi: 10.1186/s12870-021-03157-5 (PMC8359048; doi:10.1186/s12870-021-03157-5)
Supplement: Supplementary file 6 — Additional file 6: Fig. S6. Overexpression of AtTCF1 in Arabidopsis increases plant cold tolerance. [file 12870_2021_3157_MOESM6_ESM.pdf]

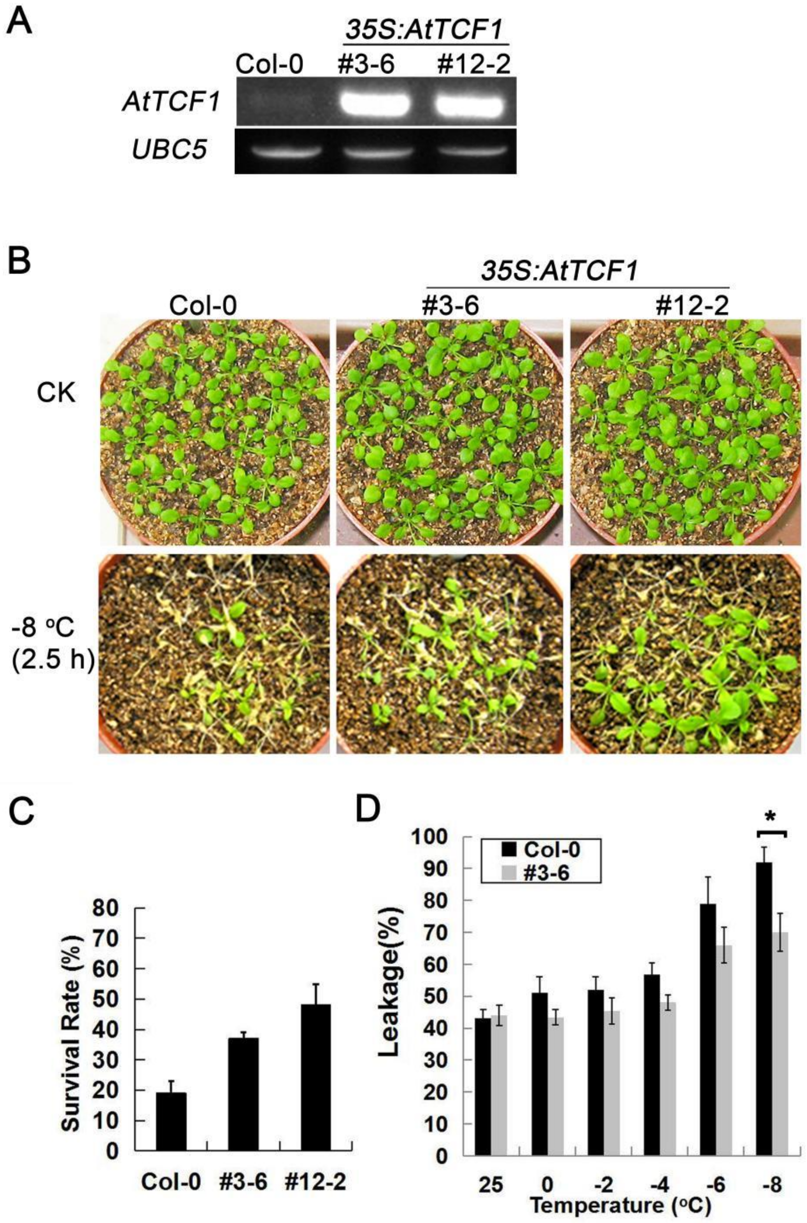

Additional file 6: Figure S6. Overexpression of *AtTCF1* in *Arabidopsis* increases tolerance to cold. **a** Expression level of *AtTCF1* in two *35S:AtTCF1* transgenic plants. *UBC5* gene was used as control. **b** Freezing assay of wild-type and two transgenic *Arabidopsis* lines (#3-6 and #12-2) at -8 ºC for 2.5 hours after cold acclimation. The photographs were taken 10 days after freezing treatment. **c** Survival rate of wild-type and *35S:AtTCF1* lines after freezing treatment from b. **d** Electrolyte leakage of wild-type and *35S:AtTCF1* lines after treated at temperatures 0 ºC, -2 ºC, -4 ºC, -6 ºC, -8 ºC for 0.5 hour. Error bars represent Mean ± SD. A paired two-sample Student's *t*-Test was performed, * indicates $P < 0.05$. These experiments were repeated for three times with the same results.
