# Supplementary material for: Enhancement of plant cold tolerance by soybean RCC1 family gene GmTCF1a
Source: BMC Plant Biol. 2021 Aug 12;21:369. doi: 10.1186/s12870-021-03157-5 (PMC8359048; doi:10.1186/s12870-021-03157-5)
Supplement: Supplementary file 7 — Additional file 7: Fig. S7. The relative expression level of AtCOR15a in wild-type and 35S:AtTCF1a transgenic Arabidopsis plants. [file 12870_2021_3157_MOESM7_ESM.pdf]

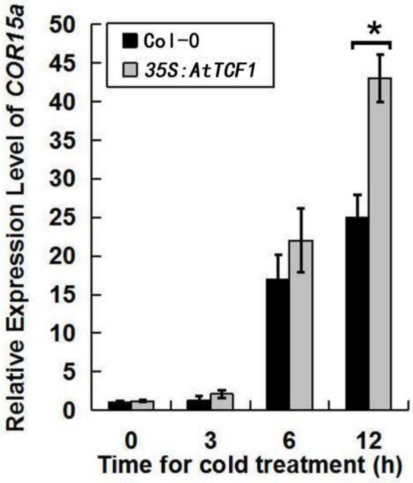

Additional file 7: Figure S7. The relative expression level of *AtCOR15a* in wild-type and *35S:AtTCF1* transgenic *Arabidopsis* plants. Three-week-old plants were subjected to low temperature (4 °C) and the samples were harvested at the indicated time points. Error bars represent the standard error of mean among three biological replicates. *, *t*-test (P < 0.05).
