# Supplementary material for: Enhancement of plant cold tolerance by soybean RCC1 family gene GmTCF1a
Source: BMC Plant Biol. 2021 Aug 12;21:369. doi: 10.1186/s12870-021-03157-5 (PMC8359048; doi:10.1186/s12870-021-03157-5)
Supplement: Supplementary file 8 — Additional file 8: Fig. S8. Domain analysis of GmTCF1s. [file 12870_2021_3157_MOESM8_ESM.pdf]

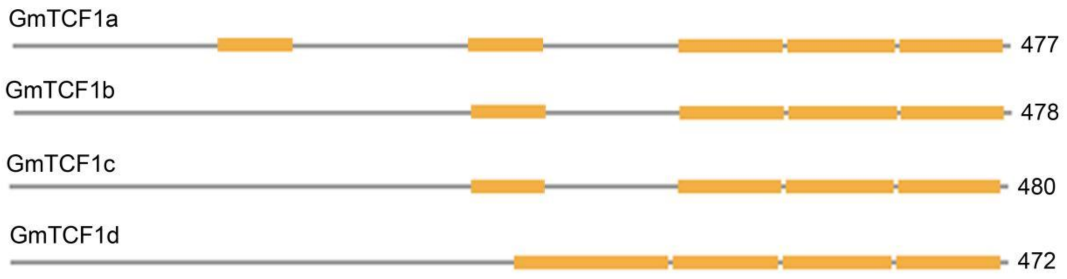

Additional file 8: Figure S8. Domain analysis of GmTCF1s. The yellow boxes indicate the RCC1 domains and position. The RCC1 domains were predicted using the SMART tool.
