# Supplementary material for: Enhancement of plant cold tolerance by soybean RCC1 family gene GmTCF1a
Source: BMC Plant Biol. 2021 Aug 12;21:369. doi: 10.1186/s12870-021-03157-5 (PMC8359048; doi:10.1186/s12870-021-03157-5)
Supplement: Supplementary file 9 — Additional file 9: Table S1. Primers used in this study. [file 12870_2021_3157_MOESM9_ESM.pdf]

Additional file 9: Table S1. Primers used in this study

Primer	Sequence
GmTCF1 Forward primer	5'-ATAATGGCGATGGAACCGAC-3'
GmTCF1 Reverse primer	5'-AACGAGGGCTCACTTGCTCT-3'
18S rRNA Forward primer	5'-CCTTGCTTGTTGCTTTACTAAAT-3'
18S rRNA Reverse primer	5'-ATGCACCTTTTCGTTTGTTTCGGAG-3'
GmTCF1 real-time Forward primer	5'-CGGTTGCCGTTAGGGTTC-3'
GmTCF1 real-time Reverse primer	5'-GCTTTGGCCTAGATCATCTGTT-3'
GmTCF1 pro:GUS Forward primer	5'-AACTGCAGCTGTCTCTGCTGTAAATCGC-3'
GmTCF1 pro:GUS Reverse primer	5'-CGGGATCCTCCCACATTCAATTCCAAAGG-3'
GFP-GmTCF1 Forward primer	5'-GGGGTACCGAATGGCCATGAATAATGGCG-3'
GFP-GmTCF1 Reverse primer	5'-CGGGATCCTCAAGTGTGGGACTCGGCCA-3'
GmTCF1 Overexpression Forward primer	5'- GCTCTAGAATGGCCATGAATAATGGCG-3'
GmTCF1 Overexpression Reverse primer	5'- GGGGTACCTCAAGTGTGGGACTCGGC-3'
CBF1 Forward primer	5'-CTTCGCTGACTCGGCTTG-3'
CBF1 Reverse primer	5'-CCACCATCGTCTCCTCCA-3'
CBF2Reverse primer	5'-GAGGATTTGGCTCGGGAC-3'
CBF2Forward primer	5'-TGGCACAGGTTGATTCCG-3'
CBF3 Reverse primer	5'-TGAGATGTGTGATGCGACG-3'
CBF3 Forward primer	5'-TTAGCCAACAAACTCGGCA-3'
COR15a Reverse primer	5'-CGCTAAAGGTGACGGCAA-3'
COR15a Reverse primer	5'-CCCAATGTATCTGCGGTTTC-3'
COR47 Forward primer	5'-GAGCGATGAAGAAGGTGAGG-3'
COR47 Reverse primer	5'-CGGGATGGTAGTGGAAACTG-3'
RD29a Forward primer	5'-GAAGATGATGATGATGACGAGC-3'
RD29a Reverse primer	5'-TCAGTGGGTTTGGTGTAATCG-3'
